# Enhanced YOLOv5s-Based Algorithm for Industrial Part Detection

**DOI:** 10.3390/s24041183

**Published:** 2024-02-11

**Authors:** Yingjian Fang, Qingxiao Wu, Sicong Li, Jian Guan, Yunge Cui

**Affiliations:** 1 Key Laboratory of Opto-Electronic Information Processing, Chinese Academy of Sciences, Shenyang 110016, China; fangyingjian@sia.cn (Y.F.);; 2 Shenyang Institute of Automation, Chinese Academy of Sciences, Shenyang 110016, China; 3 Institutes for Robotics and Intelligent Manufacturing, Chinese Academy of Sciences, Shenyang 110169, China; 4 University of Chinese Academy of Sciences, Beijing 100049, China

**Keywords:** object detection, YOLOv5s, feature fusion, industrial parts

## Abstract

In complex industrial environments, accurate recognition and localization of industrial targets are crucial. This study aims to improve the precision and accuracy of object detection in industrial scenarios by effectively fusing feature information at different scales and levels, and introducing edge detection head algorithms and attention mechanisms. We propose an improved YOLOv5-based algorithm for industrial object detection. Our improved algorithm incorporates the Crossing Bidirectional Feature Pyramid (CBiFPN), effectively addressing the information loss issue in multi-scale and multi-level feature fusion. Therefore, our method can enhance detection performance for objects of varying sizes. Concurrently, we have integrated the attention mechanism (C3_CA) into YOLOv5s to augment feature expression capabilities. Furthermore, we introduce the Edge Detection Head (EDH) method, which is adept at tackling detection challenges in scenes with occluded objects and cluttered backgrounds by merging edge information and amplifying it within the features. Experiments conducted on the modified ITODD dataset demonstrate that the original YOLOv5s algorithm achieves 82.11% and 60.98% on mAP@0.5 and mAP@0.5:0.95, respectively, with its precision and recall being 86.8% and 74.75%, respectively. The performance of the modified YOLOv5s algorithm on mAP@0.5 and mAP@0.5:0.95 has been improved by 1.23% and 1.44%, respectively, and the precision and recall have been enhanced by 3.68% and 1.06%, respectively. The results show that our method significantly boosts the accuracy and robustness of industrial target recognition and localization.

## 1. Introduction

Object detection plays an important role in the field of computer vision and is widely used in the fields of vehicle detection and pedestrian monitoring. In recent years, with the rapid development of deep learning technology, object detection has been widely applied. Especially in the field of modern industrial production, with the rise of intelligent and automated production, object detection has become more and more important. However, in industrial settings, there are a unique set of challenges on traditional object detection techniques.

Challenges in industrial production environments mainly include the following aspects: first, the quality of acquired images is negatively affected often due to poor lighting conditions, such as weak lighting in production lines or baskets. Secondly, industrial parts are diverse and have different shapes, making it more difficult to accurately identify them from images. The more challenging is that the pose of these parts in the basket varies greatly, and the same workpiece presents different characteristics in images due to different placement angles. In addition, industrial targets usually lack obvious texture and color information, which are key features commonly used by traditional object detection methods to distinguish objects, and their absence increases the difficulty of detection. Finally, due to the prevalence of random stacking and occlusion between industrial parts, the feature information of the target is further reduced, which not only reduces the accuracy of the detection, but also increases the complexity of the algorithm.

To address these challenges, this study proposes an improved algorithm based on the YOLOv5s model. The introduced C3_CA attention mechanism decomposes the channel attention into two one-dimensional feature encoding processes, which aggregate features along two spatial directions, respectively. In this way, remote dependencies can be captured along one spatial direction, while precise location information can be preserved along another spatial direction. Then, the generated feature maps are encoded into a pair of direction-aware and location-sensitive attention maps, which can be complementarily applied to the input feature map to enhance the representation of the attention object, so that more attention is paid to the characteristics of industrial targets, the background information features are ignored, and the influence of background and mutual occlusion is better distinguished. We also design a cross-layer feature fusion method (CBiFPN). Since feature information will inevitably cause the loss of feature information in the generation and transmission process of the feature pyramid, we effectively reduce the loss of information by optimizing the transmission and fusion of low-level and high-level information in the network, and fuse low-level edge information and high-level semantic information. This method improves the feature expression ability and makes full use of the feature information of industrial targets to overcome the shortcomings caused by the lack of texture color information. In order to improve the recognition rate of large targets and enhance the ability of the model to distinguish between background and industrial targets, this study introduces the Edge Detection Head (EDH). By fusing the edge information into the features, the proposed mechanism can retain the original features while strengthening the attention to the edge information. This strategy helps to suppress unnecessary background information and strengthen the feature expression of the target. By making full use of edge information, this study aims to improve the accuracy of detection.

The following work of this paper is arranged as follows: Section 2 will introduce the related work of object detection algorithms and feature fusion algorithms. Section 3 illustrates the algorithm improvement strategy proposed in this paper. Section 4 describes the experimental setup, datasets used, experimental results, and comparisons with other algorithms. Finally, Section 5 summarizes the main findings of this study and discusses directions for future work and possible improvements.

## 2. Related Works

### 2.1. Object Detection Algorithm

Object detection technology is a crucial aspect in the field of computer vision research, and its related technologies are extensively used in various aspects of production and daily life. Recently, with the rapid development of convolutional neural networks [1], object detection technology based on deep learning has advanced swiftly. Based on varying detection concepts, deep learning-based object detection algorithms can be classified into two categories: two-stage detection and one-stage detection.

#### 2.1.1. Two-Stage Algorithm

The two-stage algorithm, also known as the candidate region-based algorithm, initially generates candidate regions, followed by classification and bounding box refinement of these regions. In 2014, Ross Girshick et al. proposed the R-CNN [2] algorithm, which generates candidate boxes through selective search, extracts features using AlexNet, and classifies and predicts with a support vector machine. However, R-CNN suffers from significant overlap in the generated candidate boxes and lacks end-to-end operation capability, which adversely affects computation time. In the same year, Kaiming He et al. introduced the SPPNet [3] network, utilizing feature maps from different scales of the spatial pyramid for pooling, thereby enhancing detection speed. However, it still does not implement end-to-end operation and requires multi-stage training. In 2015, Ross Girshick et al. proposed the Fast R-CNN [4] algorithm, introducing ROI Pooling and a multi-task loss function. This achieved end-to-end training, improved the mean average precision (mAP) by 70%, and increased the speed by ninefold. Also in 2015, Shaoqing Ren et al. introduced Faster R-CNN [5]. This model’s key innovation is its region proposal network, enabling the network to self-learn in generating high-quality region proposals, thus further enhancing the speed and accuracy of object detection. Since then, various improved versions of the two-stage algorithm have been proposed and widely used in industrial production. For example, in 2022, Wei Li et al. proposed the WearNet [6] lightweight network based on CNN, which is used for scratch detection of contact sliding surfaces and significantly reduces the number of parameters in the model while ensuring the classification accuracy and recognition rate.

#### 2.1.2. One-Stage Algorithm

The one-stage algorithm performs object classification and bounding box regression simultaneously as the network generates candidate regions. Compared to the two-stage algorithm, the one-stage algorithm is quicker but slightly less precise. The YOLO (You Only Look Once) series of algorithms are renowned for their speed and effectiveness. The initial version of YOLO, YOLOv1 [7], was proposed by Joseph Redmon et al. in 2016. Its core innovation lies in treating object detection as an integrated regression problem, mapping directly from image pixels to bounding box coordinates and class probabilities, significantly enhancing processing speed. In 2017, Redmon and Farhadi introduced YOLOv2, also known as Darknet-19 [8], enhancing the detection of small-scale objects through the introduction of anchor boxes and achieving more efficient use of convolutional network features. In 2018, they proposed YOLOv3 [9], significantly enhancing detection accuracy with multi-scale detection and depthwise separable convolution, while maintaining high speed. Recently, YOLOv4 [10] was introduced by Alexey Bochkovskiy in 2020, followed by YOLOv5 [11] by Glenn Jocher in the same year. These enhancements have further optimized the network structure and training strategy, improving performance across various standard datasets. YOLOv6 [12] and YOLOv7 [13], the latest in the series, were introduced by different teams in 2021 and 2022, and continue to strike a new balance between accuracy, speed, and model size. The ongoing development and innovation within the YOLO algorithm family not only propel the advancement of one-stage object detection technology, but also offer a robust tool for real-time applications.

### 2.2. YOLOv5 Algorithm

**Figure 1:**
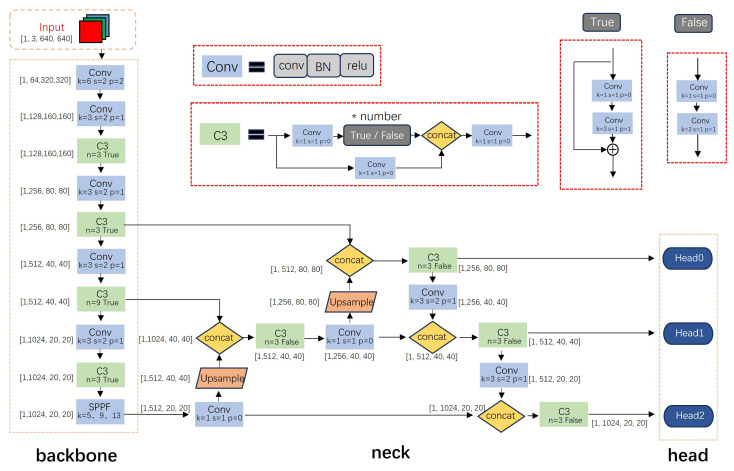
YOLOv5s network structure.

The YOLOv5 algorithm excels in both comprehensive detection accuracy and speed. The fundamental process of the YOLOv5 algorithm involves sending a set of images to the input end and applying Mosaic data enhancement [10] to enrich the dataset and improve detection effectiveness. The YOLOv5s network structure is shown in Figure 1. Subsequently, it is directed to the Backbone, where the CBH module (comprising convolutional layers, batch normalization layers, and activation functions) and CSP module [14] are employed for feature extraction, yielding feature maps of various levels. Next, it is fed into the feature fusion network (Head), which combines PAN [15] with FPN [16] for feature fusion, merging high-level semantic information with low-level texture and color information. Ultimately, three distinct feature maps of dimensions 20 × 20, 40 × 40, and 80 × 80 are generated to predict large, medium, and small objects, respectively. These feature maps are then passed to the detection Head (Head) for confidence calculation and bounding box regression, followed by post-processing involving thresholding and non-maximum suppression (NMS [17]). YOLOv5 comprises four model variants: YOLOv5s, YOLOv5m, YOLOv5l, and YOLOv5x. These four models exhibit variations in network depth and channel number. The detection accuracy of these models steadily is improved, albeit at the cost of a gradual reduction in processing speed.

### 2.3. Multi-Scale Feature Fusion

Object detection algorithms based on deep learning typically employ convolutional layers to extract features, making the receptive field size crucial for detection accuracy. Consequently, researchers combine low-resolution feature maps with large receptive fields and high-resolution feature maps with small receptive fields, a technique known as multi-scale feature fusion, to enhance object detection accuracy.

**Figure 2:**
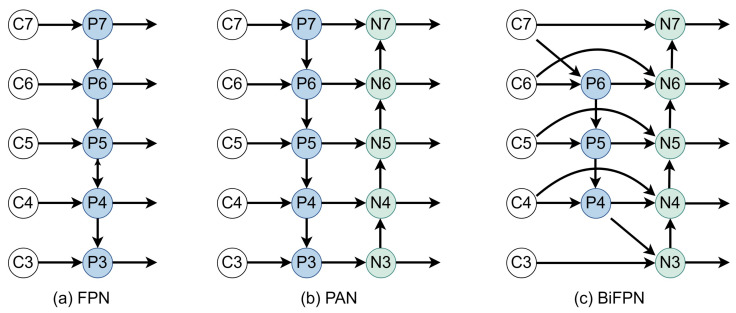
Common feature fusion structures.

An image pyramid is utilized to generate features at various scales, with each level’s feature maps preserving substantial semantic information. Nonetheless, this approach necessitates the independent extraction of features from images of different scales, incurring significant resource overhead and reducing inference speed. Wei Liu and colleagues introduced the SSD [18] algorithm, aiming to enhance inference speed by leveraging feature maps generated at various scales during convolution. The introduction of the Feature Pyramid Network (FPN) [16] addresses the challenge of separately extracting feature maps at different scales. The FPN architecture employs the top-down path of the convolution process, depicted in Figure 2, with the feature output of each level denoted as Ci. Concurrently, the high-level feature Pi is upsampled, and subsequently, high-level feature information is integrated with low-level feature information. PANet [15] introduces a bottom-up path following the top-down path of FPN, addressing the deficiency of semantic information in low-level features within the FPN architecture. The concept of BiFPN [19] is essentially derived from the notion of path-enhanced PANet, involving another round of feature fusion and redundancy removal. Additionally, different features may contribute differently, prompting the weighting of input features.

## 3. Industrial Object Recognition Models and Algorithms

### 3.1. Industrial Object Detection Model

**Figure 3:**
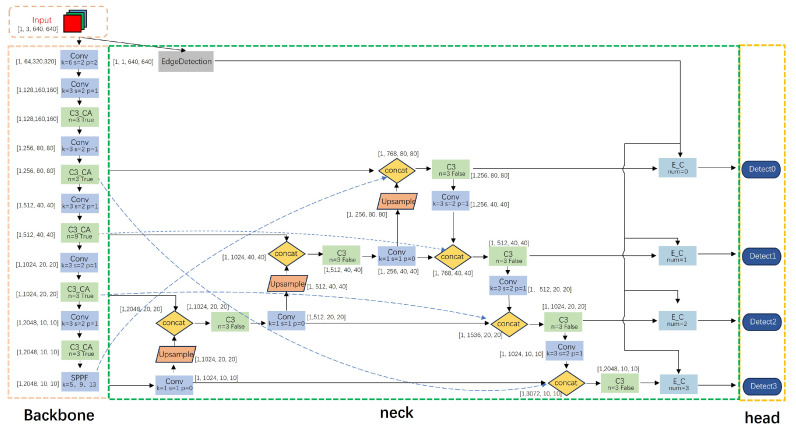
Improved YOLOv5s network structure.

In this section, we propose an improved industrial target detection model based on YOLOv5s. Through our improved model, we can identify and locate the type and location of industrial parts more quickly and accurately, facilitating subsequent industrial production. Our proposed improved YOLOv5s model framework is depicted in Figure 3. It consists of three primary components: the backbone, the neck, and the prediction heads. The backbone network is primarily composed of the C3_CA module and Conv module, effectively directing the model’s attention to the industrial part target and distinguishing it from the cluttered background to further extract features. We introduce the edge detection module to combine the acquired edge information with the original feature information. This addresses the significant shortage of industrial target detection information. Utilizing the BiFPN algorithm, we introduce a cross-feature fusion algorithm (CBiFPN) to further enhance the fusion of high-level and low-level features. Additionally, to further enhance recognition accuracy, the model employs four detection heads to improve the detector’s accuracy in detecting targets of various sizes.

### 3.2. The Design of Backbone

The backbone network is used to extract image features. Its primary function is to transform the original input image into feature maps of varying sizes and channel numbers, aiding in subsequent object detection. The original YOLOv5’s backbone layer comprises three parts: the Conv module (conv + BatchNorm + SiLU), C3 module, and SPPF module. In the backbone network, the Conv module alters the size and channel count of features to aid the C3 module in feature extraction. The basic concept of the C3 module is derived from CSPNet. It is a crucial component of the YOLOv5 network, primarily increasing the network’s depth and receptive field, thereby enhancing the C3 module’s feature extraction capabilities. The SPPF module pools multi-scale features and extracts global context features without altering the feature map’s size.

However, in industrial target detection, the workpiece’s arbitrary positioning within the material frame or on the production line results in different view features from each direction. Furthermore, considering the poor light environment in actual industrial production, the existence of mutual occlusion between industrial targets, incomplete feature extraction, and the low degree of differentiation between targets and backgrounds, it is not easy to detect targets. We hope to extract the target features more fully and distinguish the target from the background more easily, so as to improve the detection accuracy of the target. We propose the C3_CA module. This module enhances the feature extraction capability of the network by combining the attention mechanism. This module can capture not only channel information, but also direction perception and position perception information, which can help the model locate and identify the area of interest more accurately.

**Figure 4:**
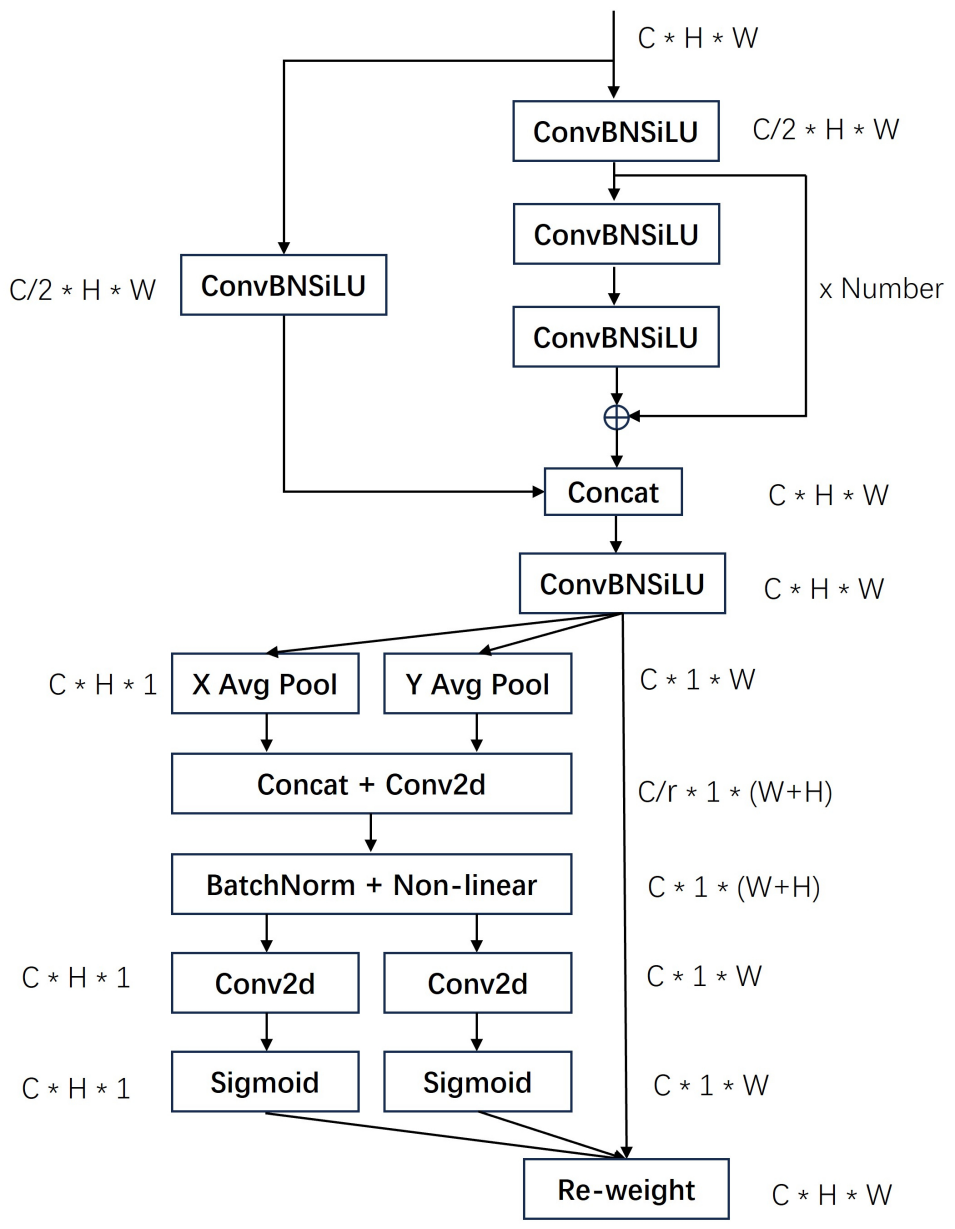
C3_CA module structure.

Common channel attention mechanisms, like CBAM and SE, typically utilize global maximum pooling or average pooling to obtain channel attention. However, this approach can lead to a loss of spatial information. Consequently, the C3_CA module integrates the spatial channel attention mechanism CA (coordinated attention) into the foundational C3 [20] module. The structure of the C3_CA module is depicted in the accompanying Figure 4. The structure of the C3_CA module is depicted in the accompanying figure. Initially, the C3_CA module conducts feature extraction on the input features. Subsequently, the feature map undergoes decomposition via global average pooling across both width and height dimensions. This process yields a direction-aware attention map in these two directions, preserving spatial dependence in one direction and precise positional information in the other. This technique is beneficial for enhancing the recognition and positioning of industrial targets. The pooling formula is as follows:

(1) $$Z_{c}^{h}\left(h\right)=\frac{1}{W}\sum_{0\leq i<W}X_{c}\left(h,i\right)$$

(2) $$Z_{c}^{w}\left(w\right)=\frac{1}{H}\sum_{0\leq j<H}X_{c}\left(w,j\right)$$

**Figure 5:**
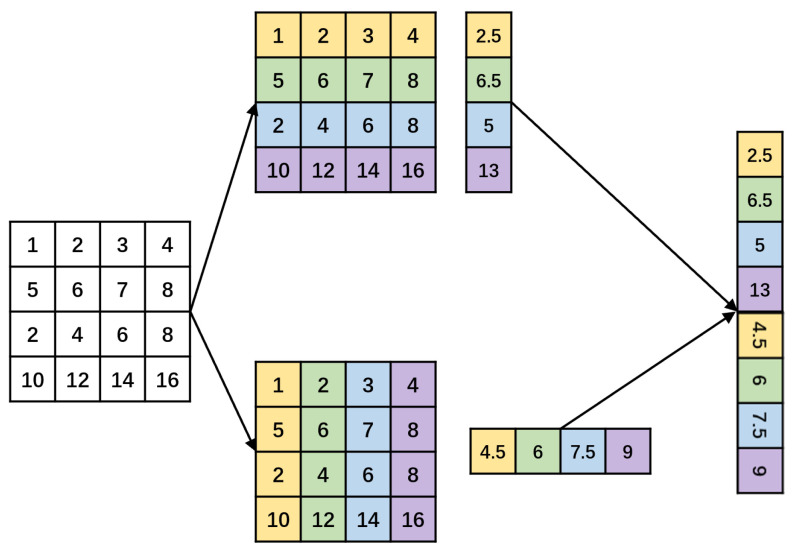
Example of global average pooling decomposition.

Consequently, by pooling the feature maps of dimensions [C, H, W], we acquire feature maps with dimensions [C, H, 1] in the width direction and [C, 1, W] in the height direction. These resultant feature maps undergo a transformation where the width and height are transposed to the last dimension, facilitating concatenation. The feature operation is shown in Figure 5. Following this, the feature undergoes convolution, normalization, and an activation function, after which it is split in the last dimension into [C, 1, W] and [C, 1, H]. Subsequent to another round of convolution and the application of a sigmoid activation function, we derive the attention vectors corresponding to both width and height dimensions.

### 3.3. CBiFPN Feature Fusion Path

**Figure 6:**
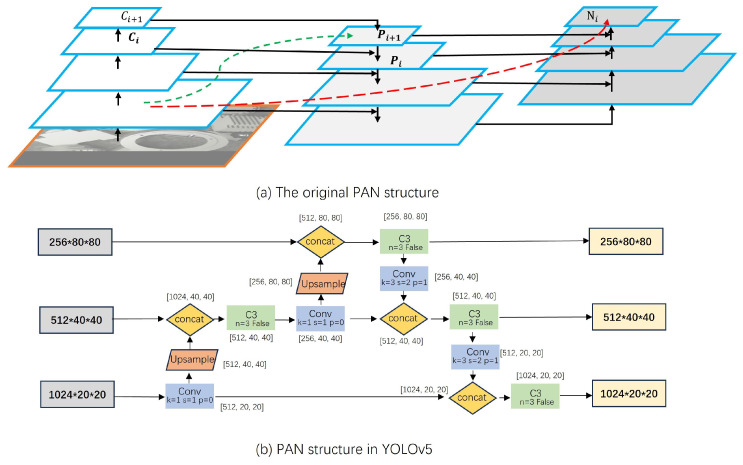
PANet network structure. Figure (**a**) shows the original PANet. Figure (**b**) shows the PANet based on YOLOv5.

In object detection, the Neck module typically combines feature maps from different levels, creating multi-scale feature maps that enhance detection accuracy. YOLOv5 employs a feature fusion module called PANet as its Neck module. The network structure and implementation of PANet in YOLOv5 are illustrated in Figure 6. PANet comprises two components: a bottom-up part and a top-down part. In the bottom-up process, the feature map from the last layer is upsampled and fused to yield more informative features. This operation is repeated until completion, mirroring the top-down process. Ultimately, the feature maps from both the top-down and bottom-up processes are combined to produce the final feature map for object detection. The 1024 × 20 × 20 feature maps generated in YOLOv5 possess abundant high-level semantic information, offering abstract features conducive to the classification of industrial parts. However, the extensive loss of detailed information during continuous downsampling and convolution hinders precise positioning. Although PANet fuses low-level detail and high-level semantic information, this results in information loss during network convolution kernel sampling. Furthermore, the shortcut operation represented by green and red dotted lines in the original PANet network is omitted. Consequently, this is disadvantageous for industrial target detection in environments with complex backgrounds, significant occlusion, and a severe lack of color and detailed information.

**Figure 7:**
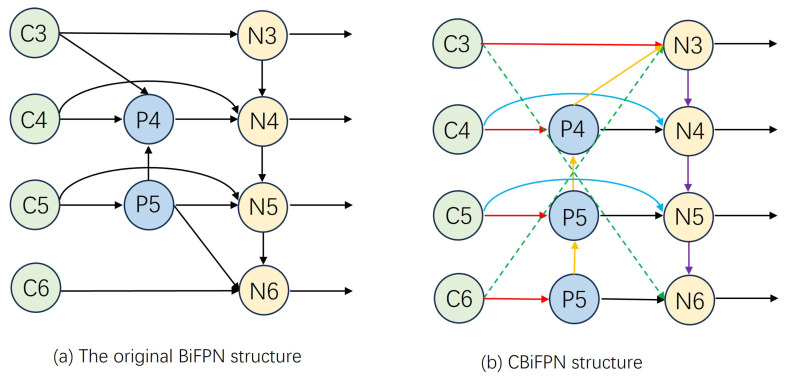
CBiFPN network structure.

In the process of industrial target detection, there are different degrees of mutual occlusion between industrial parts, the background environment is messy, the color of industrial parts is gray, and the texture information is missing. These challenges bring great challenges to the target detection in industrial scenes. Therefore, we hope to make full use of the existing feature information of industrial targets to detect and reduce the loss of target features in the network. Compared to PANet, the BiFPN network structure more effectively utilizes feature information across different scales. However, it does not optimally exploit high-level semantic information and low-level detail. Consequently, we introduce the CBiFPN network, an adaptation of the BiFPN network. The structure of this network is depicted in Figure 7.

Specifically, the feature dimensions of the outputs from C3 to C6 are assumed to be 256 × 8 × 80, 512 × 40 × 40, 1024 × 20 × 20, and 2048 × 10 × 10, respectively. Using N3 as an example, the P4 feature undergoes convolution and upsampling, with the channel number and size adjusted to match the 256 × 80 × 80 dimensions of the C3 feature. The detection head size is set at 256 × 80 × 80. A cross-connection between C6 and N3 is introduced to modify the C6 feature map. An interpolation operation adjusts the size, followed by a convolution operation to increase the channel count. The modified C6 feature map, the adjusted P4 feature map, and the C3 feature map are concatenated to create a feature map sized 768 × 80 × 80. Subsequently, the C3 module reduces the channel number to 256. Therefore, we not only realized the bidirectional connection of features from top to bottom and from bottom to top, but also avoided the loss of feature information in the bidirectional connection process through cross-layer cross-connection, realized the integration of high-level semantic information and low-level feature information, and made full use of the existing feature new information of industrial parts.

### 3.4. Edge Detection Head

**Figure 8:**
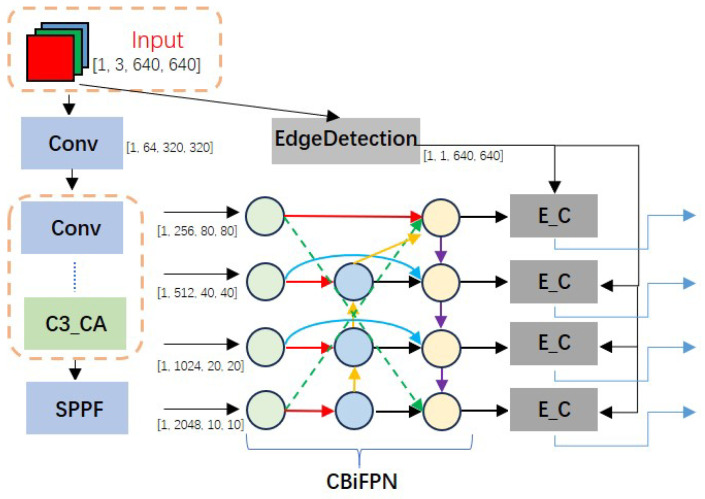
Module connection diagram.

The detection of industrial targets faces numerous challenges, including background clutter, significant occlusion, low brightness contrast, and a lack of distinct texture and color information. In practical detection, edge information is very important. Therefore, it is hoped that we can make full use of the edge information of the target to overcome the difficulties such as background clutter and lack of mutual blocking feature information of the target. In addition, the low-level edge feature information extracted by the backbone from the original image will have a certain loss in the network, so we put forward the EdgeDetectionHead (EDH) module. The process diagram is shown in Figure 8. First, EDH submodule EdgeDetection was used to obtain the edge probability graph of the original feature, and then EDH submodule E_C was used to fuse the edge probability graph obtained by CBiFPN to strengthen and highlight the edge features in the feature. This improved the contribution of edge information to detection features and provided more available features for subsequent industrial target detection tasks, thus improving the recognition accuracy. In addition, we also generated 10 × 10 feature maps for the detection of large-scale targets to improve the recall degree of target detection.

**Figure 9:**
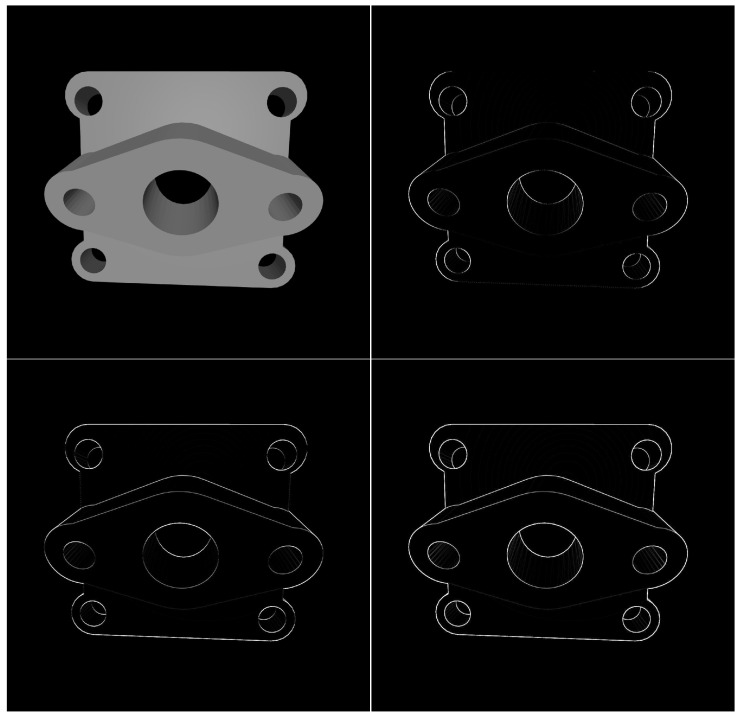
Sobel edge detection. (**Top left**): original image of the workpiece; (**top right**): horizontal edge detection image; (**bottom left**): vertical edge detection image; (**bottom right**): full edge detection map.

Prior to edge feature extraction, the incoming image to the network is converted into a grayscale format. The edge extraction algorithm employs the Sobel edge detection method. As the image is two-dimensional, it requires derivative computation in both horizontal and vertical directions. Edges in the vertical direction are characterized by larger partial derivatives in the horizontal direction, while edges in the horizontal direction exhibit larger partial derivatives in the vertical direction. As shown in Figure 9, it is necessary to calculate the gradients $G_{x}$ and $G_{y}$ across the last two dimensions, width (W) and height (H), of the image tensor *I* input into the network, followed by taking their absolute values. The final edge probability map is produced by combining the edge intensities in both the horizontal and vertical directions and applying the sigmoid function to them. The formula is as follows:

(3) $$G_{x}=\left[\begin{array}{ccc} -1 & 0 & +1\\ -2 & 0 & +2\\ -1 & 0 & +1 \end{array}\right]*I$$

(4) $$G_{y}=\left[\begin{array}{ccc} -1 & -2 & -1\\ 0 & 0 & 0\\ +1 & +2 & +1 \end{array}\right]*I$$

(5) $$G=\sqrt{G_{x}^{2}+G_{y}^{2}}$$

Considering the characteristics of the large size of industrial targets, to improve the detection accuracy of these targets, we have added a large target detection head with 1010 receptive fields to the existing three detection heads. We have designed feature maps of sizes 10 × 10, 20 × 20, 40 × 40, and 80 × 80 to detect extra-large, large, medium, and small targets, respectively. Additionally, we introduced new anchors of sizes [300, 320, 350, 400, 550, 600]. To match these four sets of feature map sizes, we resize the obtained edge detection maps and perform element-wise multiplication of the adjusted edge maps with each set of feature maps. This approach effectively highlights the edge information in the features. Finally, the original feature map is combined with the enhanced edge version. This not only retains the original features but also emphasizes the edge information, thereby enhancing the overall feature representation ability in the subsequent processing stages.

## 4. Experiments

### 4.1. Experimental Environment

The study was conducted using the ultralytics/YOLOv5 framework on a Windows 10 Professional (64-bit) system. The hardware configuration featured an Intel Core i7-8700 CPU and an NVIDIA GeForce RTX 3060 Laptop GPU with 12 GB VRAM, backed by 32 GB of system RAM. On the software front, Python 3.9.18 and PyTorch 2.0.1+cu118 were utilized. Key experimental parameters included a learning rate (lr0) of 0.01, training epochs set at 100, batch size maintained at 8, and an image resolution of 640 × 640 pixels.

### 4.2. Datasets

**Figure 10:**
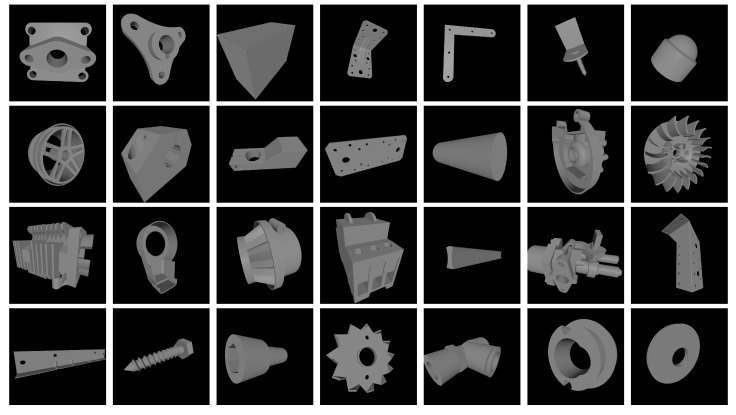
28 industrial parts.

To evaluate the performance of the improved algorithm for industrial object detection, this study utilizes the MVTec ITODD dataset, which has been modified and filtered to create a dataset suitable for our experiments. The MVTec ITODD dataset comprises data on 28 different objects (such as adapter_plate_square, adapter_plate_traingular, clamp_big, etc.) distributed across more than 3500 different scenes (environments with different backgrounds, different shades, and different brightness and contrast), enabling research in the domains of 3D object detection and pose estimation. Sample pictures of 28 categories of these targets are displayed in Figure 10. The dataset comprises 50,000 RGB images and their corresponding annotation files. Initially, we adjusted the format of the annotation file to meet the annotation format requirements of the YOLO detection algorithm. Subsequently, we filtered the images to eliminate those with exceptionally low brightness, poor contrast, and significant occlusion. Lastly, we chose 4000 images from the original dataset for training, along with 640 images for validation experiments and another 640 images for testing. The data enhancement technique uses YOLO’s default Mosaic approach.

**Figure 11:**
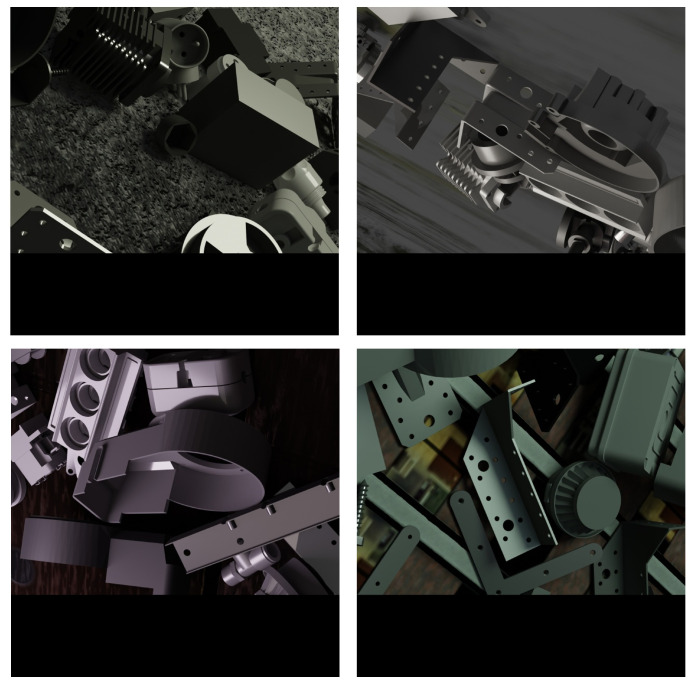
Modified ITODD dataset.

Figure 11 depicts sample pictures employed in this study, showcasing the characteristics of our filtered dataset. The size of the original image is 1280 × 960. To maintain the shape and relative position of the object in the image unchanged, we scale the original image to the size of 640 × 480 using uniform scaling. Afterwards, we added black pixels to the bottom of the image to achieve a final image size of 640 × 640. This processing helps preserve the geometric features of the target while meeting the image size requirements necessary for the experiment.

**Figure 12:**
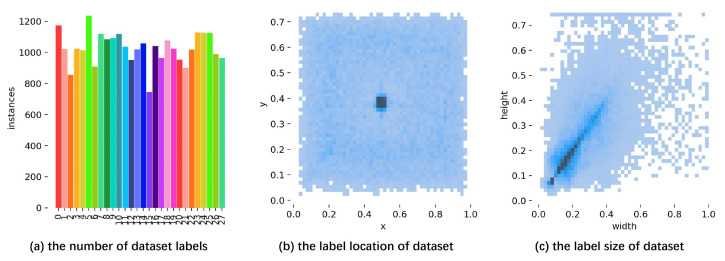
Visualization results of training set label attributes.

In Figure 12a, the distribution of the number of samples of each class in the training set is shown. It is worth noting that the number of samples in each category is roughly equal, ensuring the balance of the dataset. In Figure 12b, we present the location distribution of industrial targets in the image and observe that these targets are mainly concentrated in the central region of the image. Finally, in Figure 12c, we show the height and width of the target annotation box; this information shows the size scale of the target with respect to the whole image.

### 4.3. Evaluation Criterion

In this study, precision (P), recall (R), and mean average precision (mAP) are used as evaluation indicators to evaluate the experiment. The corresponding formulas are as follows:

(6) $$Precision=\frac{TP}{TP+FP}$$

(7) $$Recall=\frac{TP}{TP+FN}$$

(8) $$mAP=\frac{1}{n}\sum_{i=1}^{n}AP_{i}$$

In these formulas, TP (True Positives) represents the number of instances correctly classified as positive class, FP (False Positives) represents the number of instances incorrectly classified as positive class, and FN (False Negatives) represents the number of positive class instances incorrectly classified as negative class. The mean average precision (mAP) refers to the average of the average precision (AP) values across all categories and query images. The AP for each category is calculated as the mean of the precision of the model at different recall thresholds. The mAP@0.5 and mAP@0.5:0.95 are variants of mAP computed under specific Intersection over Union (IoU) threshold conditions.

### 4.4. Experimental Results and Analysis

Table 1 shows the performance comparison between YOLOv5s and the improved algorithm of this study. Compared with the original YOLOv5s model, the improved algorithm proposed in this study achieves a 3.68% improvement in precision, reaching 90.48%. In terms of recall, it is increased by 1.06% to 75.81%. In addition, the performance of the model on mAP@0.5 is increased by 1.23% to 83.34% and on mAP@0.5:0.95 is increased by 1.44% to 62.42%. These results clearly prove the significant improvement of the comprehensive performance of the improved algorithm.

**Figure 13:**
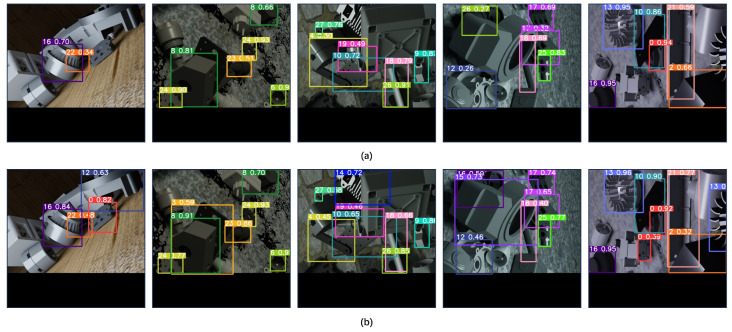
Comparison of industrial target detection results. (**a**) Original YOLOv5s algorithm. (**b**) The proposed algorithm.

Then, we provide a visual analysis of the application effects of the proposed algorithm in an industrial target detection task. In this study, five images were randomly selected from the test set for the detection task experiment. By comparing the detection results of the original YOLOv5s model with those of the improved YOLOv5 model, we find that the improved model generally exhibits higher confidence. Specifically, the detection results of the original model are shown in Figure 13a, while those of the improved model are displayed in Figure 13b.

Experimental results demonstrate that, compared to the original YOLOv5s algorithm, our proposed improved algorithm offers superior performance in industrial target detection. In particular, the C3_CA attention module, integrated into the algorithm, more effectively focuses on the target and distinguishes the background in environments with complex backgrounds, thereby significantly enhancing detection accuracy. Additionally, the feature fusion component further boosts detection reliability by effectively combining high-level semantic information with low-level detail information.

**Figure 14:**
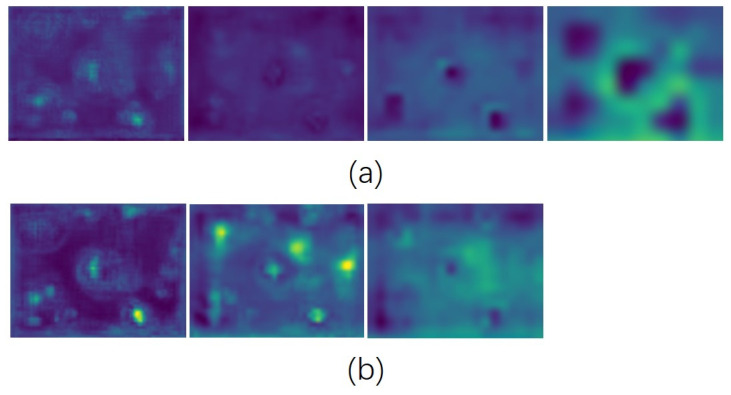
Feature map.

For the detection of large objects, our enhanced edge detection head (EDH) markedly improves the ability to detect large objects, objects in mutual occlusion, and incomplete industrial objects located at the image’s edge. The feature maps, both pre- and post-improvement of the algorithm, are displayed in Figure 14. For better observation, we have scaled the feature maps of each scale to a uniform size. Figure 14a illustrates the feature maps at each scale enhanced by the algorithm, showing clearer, more distinct, and more focused features. In contrast, Figure 14b depicts the feature map before improvement, characterized by issues such as a lack of prominent feature points, more background noise, and non-uniformity. Overall, the improved algorithm proposed in this study demonstrates significant advantages in the field of industrial target detection.

### 4.5. Comparison with Other Object Detection Algorithms

As shown in Table 2, our algorithm demonstrates superior performance across various key performance metrics. Specifically, when compared to the YOLO series algorithms YOLOv3 and YOLOv7, we first set the same batch size, learning rate, network depth, and width as YOLOv5s. Our algorithm enhances precision by 2.93 and 6.28 percentage points, respectively. Our algorithm achieves a high accuracy rate of 90.48%. In terms of recall, our algorithm outperforms YOLOv3 and YOLOv7, achieving 75.81%. On the evaluation metric mAP (mean average precision), particularly on the comprehensive performance index mAP@0.5:0.95, our algorithm attains 62.42%, significantly surpassing YOLOv3’s 59.36%, YOLOv7’s 60.59%, and Faster R-CNN’s 59.99%. This result shows the considerable enhancement in detection accuracy of our algorithm, particularly its robustness in handling objects of various scales.

Concurrently, we observe that Faster R-CNN exhibits a performance comparable to our algorithm on mAP@0.5, achieving 83.03%. This suggests that the two-stage detection method is still competitive in accuracy for certain detection tasks. Conversely, the performance of the SSD algorithm is somewhat lacking, particularly with only 50.95% on mAP@0.5:0.95. To validate the performance of our proposed algorithm in the feature fusion network, we conducted a specific comparative experiment against the YOLOv5s version augmented with the BiFPN network. The experimental results reveal that our algorithm exhibits clear advantages in all metrics, conclusively demonstrating its superiority in integrating features of different scales and adapting to complex scenes.

### 4.6. Ablation Study

In the ITODD dataset, we conducted ablation experiments on each proposed module to assess their specific impacts on model performance. The experimental results are detailed in Table 1.

The introduction of the CBiFPN module significantly enhances the accuracy of workpiece detection, achieving 87.82%, and increases mAP@0.5 to 82.44%. CBiFPN effectively addresses YOLOv5’s limitations in feature fusion with its bidirectional propagation and multi-scale fusion mechanism. By incorporating low-level feature information into high-level semantic information to compensate for the latter’s lack of detailed features and integrating high-level semantic information into low-level features, it effectively improves the accuracy and performance of industrial target detection, while fully utilizing target feature information and reducing the impact of the innate deficiency of color texture features.

By incorporating spatial information into the channel attention mechanism, the C3_CA module captures the inter-channel dependencies and effectively models location information and long-range dependence. This module not only emphasizes the importance of each channel but also accounts for the characteristics of different spatial locations, especially in complex scenes with diverse targets, thereby enriching and enhancing feature representation. Experimental results demonstrate that the C3_CA module significantly boosts the overall performance of the object detection model, increasing the precision and recall rates by 1.6% and 0.13%, respectively, and raising mAP@0.5 to 82.44%. The introduction of the EDH module significantly enhances the precision, recall, and mAP metrics of detection. By extracting the edge map of the target image and integrating it with the features in the detection head, this module not only retains the features input to the detection head but also accentuates the edge information within these features. Edge information is crucial in industrial part detection tasks. Furthermore, by generating features at four different scales—extra-large, large, medium, and small—this module effectively meets the detection needs of targets of various sizes and distances, thereby substantially improving the detection’s effectiveness and performance.

## 5. Conclusions

This study proposes an enhanced YOLOv5s-based industrial target detection algorithm to increase the accuracy of part detection in complex industrial settings. The algorithm’s performance is effectively boosted by incorporating the CBiFPN, the C3_CA attention module, and the EDH edge detection head. The CBiFPN module enables multi-scale and multi-level feature fusion, optimizing the blend of high-level semantic features with low-level detail features. This module not only fully utilizes the characteristics of industrial parts to compensate for the lack of color and texture information, but also enhances the network’s feature representation capacity, minimizes information loss, and improves target detection performance in industrial settings. When combined with the C3_CA attention module, the network’s ability to differentiate between target and background features in complex scenes with diverse targets is enhanced, leading to richer and more effective feature representation. Furthermore, the use of the EDH edge detection head not only boosts the detection capability for large targets, but also enhances detection accuracy by merging the edge information of the original image with the feature map, achieving optimized feature representation. Compared to the original YOLOv5s benchmark model, the enhanced model shows impressive performance on the ITODD dataset, with mAP@0.5 increased by 1.23%, and improvements in detection accuracy and recall rate by 3.68% and 1.06%, respectively. Improved inspection accuracy and recall of industrial parts enable more accurate identification and positioning of parts, reducing false positives and missed tests. This helps reduce the need for manual intervention and adjustment, improve the operational efficiency of the production line, reduce production interruptions and downtime, and improve production efficiency. The improvement of mAP value can help optimize the selection of models and algorithms, improve the level of automation and intelligence, and reduce the need for manual intervention and subjective judgment.

However, we anticipate some challenges in our research. Labeling large datasets for industrial production may pose difficulties. Additionally, despite the improvements made to the YOLOv5s algorithm, limitations in accuracy may still exist. For instance, the algorithm may struggle with accurate target positioning and classification in complex scenes where targets are heavily occluded or with small-sized targets. To address these challenges, we will continue to refine the algorithm, improve the precision and accuracy of target detection, and strive for continuous optimization and improvement. In future research, our focus will be on optimizing the industrial object detection algorithm based on enhanced YOLOv5s. We plan to explore and enhance feature fusion techniques to improve the algorithm’s performance in complex industrial environments. Moreover, we intend to investigate attention mechanisms and edge detection methods to further enhance detection accuracy and robustness. Through continuous experimentation and evaluation, we aim to ensure a continuous improvement in the algorithm’s performance in industrial scenarios.

## Figures and Tables

**Table 1:** Ablation experiment results.

Model	Precision	Recall	mAP@0.5	mAP@0.5:0.95
YOLOv5s	86.8	74.75	82.11	60.98
YOLOv5s + CBiFPN	87.82	74.88	82.44	61.16
YOLOv5s + C3_CA	88.40	75.11	82.67	61.45
YOLOv5s + EDH	90.16	75.24	83.22	62.17
**ours**	**90.48**	**75.81**	**83.34**	**62.42**

**Table 2:** The performance of this algorithm compared with various object detection algorithms.

Model	Precision	Recall	mAP@0.5	mAP@0.5:0.95
YOLOv3	87.55	74.07	81.04	59.36
YOLOv7	84.20	74.86	81.00	60.59
YOLOv5 + BiFPN	85.49	73.58	80.80	59.20
Faster R-CNN	-	-	83.03	59.99
SSD	-	-	79.48	50.95
**TheProposed**	**90.48**	**75.81**	**83.34**	**62.42**

## Data Availability

Data available within the article.
